# The Tumor Suppressor, P53, Decreases the Metal Transporter, ZIP14

**DOI:** 10.3390/nu9121335

**Published:** 2017-12-08

**Authors:** Ningning Zhao, An-Sheng Zhang, Aaron M. Wortham, Shall Jue, Mitchell D. Knutson, Caroline A. Enns

**Affiliations:** 1 Department of Nutritional Sciences, The University of Arizona, Tucson, AZ 85721, USA; 2 Department of Cell, Developmental, and Cancer Biology, Oregon Health & Science University, Portland, OR 97239, USA; zhanga@ohsu.edu (A.-S.Z.); worthama@ohsu.edu (A.M.W.); jues@ohsu.edu (S.J.); 3 Department of Food Science and Human Nutrition, University of Florida, Gainesville, FL 32611, USA; mknutson@ufl.edu

**Keywords:** ZIP14, p53, tumor suppressor, iron

## Abstract

Loss of p53’s proper function accounts for over half of identified human cancers. We identified the metal transporter ZIP14 (Zinc-regulated transporter (ZRT) and Iron-regulated transporter (IRT)-like Protein 14) as a p53-regulated protein. ZIP14 protein levels were upregulated by lack of p53 and downregulated by increased p53 expression. This regulation did not fully depend on the changes in ZIP14’s mRNA expression. Co-precipitation studies indicated that p53 interacts with ZIP14 and increases its ubiquitination and degradation. Moreover, knockdown of p53 resulted in higher non-transferrin-bound iron uptake, which was mediated by increased ZIP14 levels. Our study highlights a role for p53 in regulating nutrient metabolism and provides insight into how iron and possibly other metals such as zinc and manganese could be regulated in p53-inactivated tumor cells.

## 1. Introduction

Over 50% of identified human cancers are caused by mutations in the gene encoding tumor suppressor p53 [1,2,3,4]. P53 has a well-documented function as a transcription factor [5]. It also possesses transcription-independent cytosolic functions, including inhibition of autophagy [6,7,8,9], regulation of mitochondrial function [10,11] and regulation of apoptosis [12]. Inactivation of p53 contributes not only to cancer initiation and progression, but also to chemotherapy resistance [13,14]. Tumor cells are characterized by uncontrolled proliferation. Rapidly proliferating cells need considerably more nutrients than normal cells. Tumor metabolism is altered to accommodate for the increased nutrient requirement and metabolic demand. Elucidation of p53’s function in regulating nutrient transport is important to the understanding of how p53 influences tumor metabolism.

Iron, an essential nutrient, is required by nearly all cells to maintain energy metabolism, DNA synthesis and cell proliferation. Rapidly growing and frequently dividing tumor cells need considerably more iron than quiescent cells. Iron promotes tumor initiation by enhancing free radical formation and accelerates tumor growth by fostering the cell proliferation [14]. Increasing cellular iron content by repressing the iron storage protein ferritin leads to increased tumor cell proliferation [15]. Tumor cells also have elevated iron uptake to meet the increased iron need [14]. Under most physiological conditions, transferrin-bound iron (TBI) is the major iron source and is mediated through the endocytosis of transferrin (Tf)/transferrin receptor 1 (TfR1) complex [16]. Under conditions of increased iron-bound Tf (Tf-saturation) or decreased Tf availability, such as conditions in cancer patients undergoing chemotherapy, levels of plasma non-transferrin-bound iron (NTBI) will increase significantly [17,18,19,20]. NTBI augments tissue oxidative damage, promotes tumor growth, and contributes to the therapy resistance. Two iron transport proteins, ZRT/IRT-like protein 14 (ZIP14) [21,22], and divalent metal-ion transporter 1 (DMT1) [23,24,25], have been identified to mediate both TBI and NTBI uptake into cells, and both transporters localize at the plasma membrane as well as endocytic compartments [23,25,26].

Several mechanisms have been described relating p53 and iron metabolism. For instance, induction of stably transfected p53 containing a doxycycline-inducible activator in H1299 lung cancer cells reduced TfR1 levels, suggesting that p53 may promote cell cycle arrest by reducing the availability of intracellular iron [27]. Iron metabolism has been reported to modulate p53 through direct binding of heme to p53 [28]. This binding disrupts p53-DNA interactions and triggers the nuclear export and cytosolic degradation of p53. A recent study has linked an iron-dependent, non-apoptotic cell death pathway, called ferroptosis with p53, and demonstrated that p53 suppresses tumor growth at least partially through ferroptosis [29]. Interestingly, this study identified the cell-surface amino acid transporter SLC7A11 as a target gene whose expression is inhibited by p53. However, despite the reported relationships between cancer and iron biology, the effects of the tumor suppressor p53 on the cell-surface iron transport proteins, ZIP14 and DMT1, have not been investigated.

The present study aimed to test whether p53 plays a role in regulating the expression of ZIP14 and/or DMT1, thus regulating cellular non-transferrin-bound iron (NTBI) uptake. By using two cell lines, human hepatoma cells (HepG2) and human embryonic kidney cells (HEK293), which express wild-type p53, we found that knockdown of endogenous p53 increased ZIP14 levels and NTBI uptake, but did not change DMT1 levels. We demonstrated that the increased iron uptake was mediated through elevated ZIP14 expression. Mechanistically, p53 co-precipitated with ZIP14, and deprivation of p53 decreased ZIP14 ubiquitination and prevented the degradation of plasma membrane ZIP14. Decreased levels of p53 also increased ZIP14 mRNA expression. Thus, ZIP14 is a newly identified p53-regulated protein. Our results highlight the role of p53 in the regulation of nutrient transporters and provide insight into how loss of functional p53 may alter cellular iron metabolism.

## 2. Materials and Methods 

### 2.1. Cell Culture, Transfection, Stable Cell Lines, and Gene Silencing

HepG2 cells were grown in Minimum Essential Medium (MEM, Sigma, St. Louis, MO, USA) with 1× non-essential amino acids (NEAA) and 10% fetal bovine serum (FBS, Atlanta Biologicals, Flowery Branch, GA, USA). HEK 293 cells were grown in Dulbecco’s Modified Eagle’s Medium (DMEM, Sigma, St. Louis, MO, USA) with 4.5 g/L glucose, 4 mM/L-glutamine, 1 mM sodium pyruvate, and 10% FBS. All cells were maintained in an incubator at 37 °C and 5% CO_2_. Plasmids encoding wild-type (WT) human-MYC-FLAG-ZIP14 (pCMV-Entry-hZIP14) and WT human p53 (pCMV-p53-WT) were purchased from Origene (Rockville, MD, USA) and Addgene (Cambridge, MA, USA), respectively. Effectene transfection reagent (Qiagen, Germantown, MD, USA) was used for transfection. Briefly, cells were seeded at 40% confluency in 6-well culture plates or 100 mm culture dishes. Transfection began 24 h after seeding with 0.4 µg or 2 µg of plasmid DNA, 3.2 µL or 16 µL of enhancer and 10 µL or 50 µL of Effectene reagent. Transfection was carried out for 48 h before further analysis. HEK293 cells stably expressing WT human ZIP14 were generated as previously described [30]. For transfection in HepG2 cells, 1 µg or 2 µg of plasmid DNA, 8 µL or 16 µL of enhancer, and 10 µL or 50 µL of effectene reagent were used. Gene silencing was performed by using small interfering RNA (siRNA). Cells were transfected with gene specific siRNAs or a universal scrambled negative control siRNA (used at 25 nM each, Origene) by using Lipofectamine RNAiMAX reagent (Invitrogen, Waltham, MA, USA) according to the manufacturer’s reverse transfection protocol. Cells were incubated with the siRNA for 48 h before further analysis. The efficiency of p53 or ZIP14 depletion was confirmed by immunoblotting.

### 2.2. Quantitative Real-Time RT-PCR (qRT-PCR)

RNA was extracted by NucleoSpin RNA II kit (Takara, Mountain View, CA, USA) according to the manual. Complementary DNA (cDNA) was synthesized by Moloney Murine Leukemia Virus Reverse Transcriptase (M-MLV RT) (Promega, Madison, WI, USA). The qRT-PCR was performed by Applied BiosystemsViiA 7 system (Thermo Scientific, Waltham, MA, USA). Thermocycling conditions were: 50 °C for 2 min; 95 °C for 10 min; 95 °C for 15 s and 60 °C for 1 min for 40 cycles. Each 15 μL reaction contained: 1× SyberGreen Master Mix, 1 μL forward and reverse primer (5 μM each) and 2 μL of cDNA template. Primer sequences for q-RT-PCR are as follows. For human ZIP14, forward: 5′ GTC TGG CCT TTG GCA TCC T 3′, reverse: 5′-AGG GAA CAT ATC AGC CAG AGA AAT-3′; for FLAG sequence, forward: 5′-GGC CGC TCG AGC AGA AAC-3′, reverse: 5′-TCG TCG TCA TCC TTG TAA TCC AGG 3′; for β-actin, forward: 5′-GTC ATT CCA AAT ATG AGA TGC GT-3′, reverse: 5′-GCT ATC ACC TCC CCT GTG TG-3′.

### 2.3. Isolation of Plasma Membrane Protein

Plasma membrane proteins were labeled by the membrane-impermeable, thiol-cleavable biotinylation reagent, NHS-SS-biotin (Thermo Scientific, Waltham, MA, USA) and isolated by using the Streptavidin Resin (GBiosciences, St. Louis, MO, USA) as previously described [30]. Immunoblot analysis was used to detect cell-surface ZIP14. Plasma-type Na^+^, K^+^ ATPase was used as a control for plasma membrane protein.

### 2.4. CRISPR/Cas9-Mediated Generation of HEK293 Cell Line Expressing FLAG Tagged ZIP14 from Endogenous Locus

The Clustered Regularly Interspaced Short Palindromic Repeats (CRISPR)/CRISPR associated protein 9 (Cas9)-mediated genome editing approach has been described previously [31,32]. Briefly, a pair of oligos for single-guide RNA (sgRNA) targeting the stop codon region of human ZIP14 gene was cloned into the px330-U6-hSpCas9 vector for the expression of Cas9 and sgRNA (targeting vector). Single-stranded ultramer DNA oligos (ssODN) containing 3× FLAG sequence flanked by ~60 bp homologous regions were used as the homologous recombination (HR) repair template. Oligo sequences used for sgRNA and ssODN are as follows. For the pair of sgRNA, 5′-CACC GGA CAG ATC CAG ATT GGG TA 3′ and 5′-AAAC TAC CCA ATC TGG ATC TGT CC 3′; for ssODN, 5′-AAC CTG GGC CTC CTG ACT GGA TTC ACC ATC ATG GTG GTC CTC ACC ATG TAT TCA GGA CAG ATC CAG GAC TAT AAG GAC CAC GAC GGA GAC TAC AAG GAT CAT GAT ATT GAT TAC AAA GAC GAT GAC GAT AAG TAG GGC TCT GCC AAG AGC CTG TGG GAC TGG AAG TCG GGC CCT GGG CTG CCC GAT CGC CAG CCC GAG G-3′. For genotyping, forward primer: 5′ GGA AGG GCA GCA TCT TGA TTC C-3′; reverse primer: 5′-CCT CTT CCG TGG TGC ATT GTG-3′. HEK293 cells were electroporated with Neon transfection device and kit (Invitrogen, Waltham, MA, USA). The transfection complex contains a mixture of ~5 × 10^6^ cells, 2 µg of targeting vector, and 1 µM of ssODN. After transfection, cells were seeded to a six-well plate and grown for 48 h. Cells were then diluted and split into 10 cm dishes to select single cell clones. About 100 single cell colonies were screened. Successful 3× FLAG sequence knock in were confirmed by PCR, Sanger sequencing, and immunoblot analyses.

### 2.5. Immunoblotting and Immunoprecipitation

Cells were washed twice with cold PBS and lysed in NETT buffer (150 mM NaCl, 5 mM EDTA, 10 mM Tris, 1% Triton X-100 and 1× Protease Inhibitor Cocktail (Bimake, Houston, TX, USA), pH 7.4). Cell lysates were centrifuged at 10,000× *g* for 10 min at 4 °C. Nuclear and cellular debris were discarded. Protein concentrations of the post-nuclear supernatant were measured by using the *RC DC* Protein Assay (Bio-Rad, Hercules, CA, USA). Samples were mixed with 1× Laemmli buffer and incubated for 30 min at 37 °C. Proteins were separated electrophoretically on an SDS/10% polyacrylamide gel, transferred to nitrocellulose and incubated for 1 h in blocking buffer (5% nonfat dry milk in Tris-buffered saline-Tween 20, TBST). Blots were incubated for 1 h at room temperature in blocking buffer containing mouse anti-FLAG, anti-FLAG-HRP, M2 (1:10,000, Sigma, St. Louis, MO, USA), rabbit anti-DMT1 (Proteintech, Rosemont, IL, USA, 1:5000), or mouse anti-TfR1 (Thermo Scientific, Waltham, MA, USA, 1:5000). After four washes with TBST, blots were incubated with a 1:5000 goat anti-mouse secondary antibody conjugated to horseradish peroxidase (HRP, Millipore, Burlington, MA, USA). To confirm equivalent loading, blots were stripped for 15 min in Restore PLUS Western Blot Stripping Buffer (Thermo Scientific, Waltham, MA, USA), blocked for 1 h in blocking buffer, and reprobed with mouse anti-actin (Millipore, Burlington, MA, USA, 1:10,000) or rabbit anti-tubulin (Rockland, Limerick, PA, USA, 1:5000) followed by HRP-conjugated goat anti-mouse (Millipore, Burlington, MA, USA) or donkey anti-rabbit (GE Healthcare, Little Chalfont, UK) secondary antibody. For loading control of plasma membrane proteins, mouse anti-Na^+^, K^+^ ATPase antibody (1:2000, Santa Cruz, Dallas, TX, USA) followed by HRP-conjugated secondary antibodies were used. After two washes with TBST and TBS, bands were visualized by using enhanced chemiluminescence (SuperSignal West Pico, Thermo Scientific, Waltham, MA, USA) and X-ray film. For quantification, after primary antibody incubation, blots were probed with infrared fluorescent dye (IRDye 800) conjugated rabbit anti-mouse or Alexa Fluor 680 conjugated goat anti-rabbit secondary antibody (Thermo Scientific, Waltham, MA, USA) and visualized using a Licor Imaging System (LI-COR, Lincoln, NE, USA). HepG2 cell with endogenously FLAG tagged ZIP14 (HepG2-ZIP14-FLAG cells) were used for immunoprecipitation analysis. The post-nuclear supernatant fractions of the cell lysates were incubated with anti-FLAG (M2) agarose beads (Sigma, St. Louis, MO, USA) for 1 h at 4 °C. The beads were washed three times for 10 min in NETT buffer. The protein complex was eluted from the beads with elution buffer (0.5 mg/mL triple FLAG peptide in TBS with protease inhibitor). The elution sample was separated into two halves and analyzed by immunoblotting. One half was probed for FLAG-ZIP14 and Actin. Another half was probed by anti-ubiquitin and anti-p53 antibodies.

### 2.6. Cellular Iron Uptake Assay

The iron uptake analysis was performed as previously described [26]. Briefly, for non-transferrin-bound iron uptake, HepG2-ZIP14-FLAG cells grown in six-well plates were washed three times with serum free media (SFM) and incubated for 1 h in SFM. Cells were incubated with 2 µM ^55^Fe (ferric-citrate) for 2 h and then washed three times with cell membrane-impermeable iron chelator solution to remove cell surface-bound iron. Cells were solubilized with lysis buffer (0.1% Triton X-100, 0.1% NaOH) and cell-associated radioactivity was determined by a scintillation counter. Iron uptake was calculated as cpm/mg of protein and expressed as percent of control.

### 2.7. Measurement of Iron Levels by Inductively Coupled Plasma Mass Spectrometry (ICP-MS)

The cellular iron level was determined by ICP-MS. Briefly, HepG2 cells were transfected with p53-specific siRNA or negative control siRNA for 48 h in a six-well plate. Cells were washed four times with ice-cold PBS-EDTA (2 mM) and solubilized with 400 µL lysis buffer (0.2 M NaOH, 0.2% SDS). The protein concentration was determined by using the *RC DC* Protein Assay (Bio-Rad). The cell lysates were digested in nitric acid at a concentration of 12%. Digestion was carried out at 85 °C for 16 h and 95 °C for an additional 2 h. The digested lysates were diluted in Milli-Q H_2_O to a final concentration of 1% nitric acid. The iron concentration was measured by an Agilent 7700 Series ICP-MS instrument. The ICP-MS analyses were performed by the Arizona Laboratory for Emerging Contaminants (ALEC) at the University of Arizona.

### 2.8. Statistical Analysis

Data were analyzed by one-way ANOVA or unpaired t-test with GraphPad Prism software, version 5 (GraphPad Software, La Jolla, CA, USA). Tukey’s post hoc comparisons tests were performed with multiple comparisons. *p*-values < 0.05 were considered to be statistically significant.

## 3. Results

### 3.1. Suppression of P53 Increases Endogenous Levels of ZIP14, but Not DMT1 in HepG2 Cells

**Figure 1:**
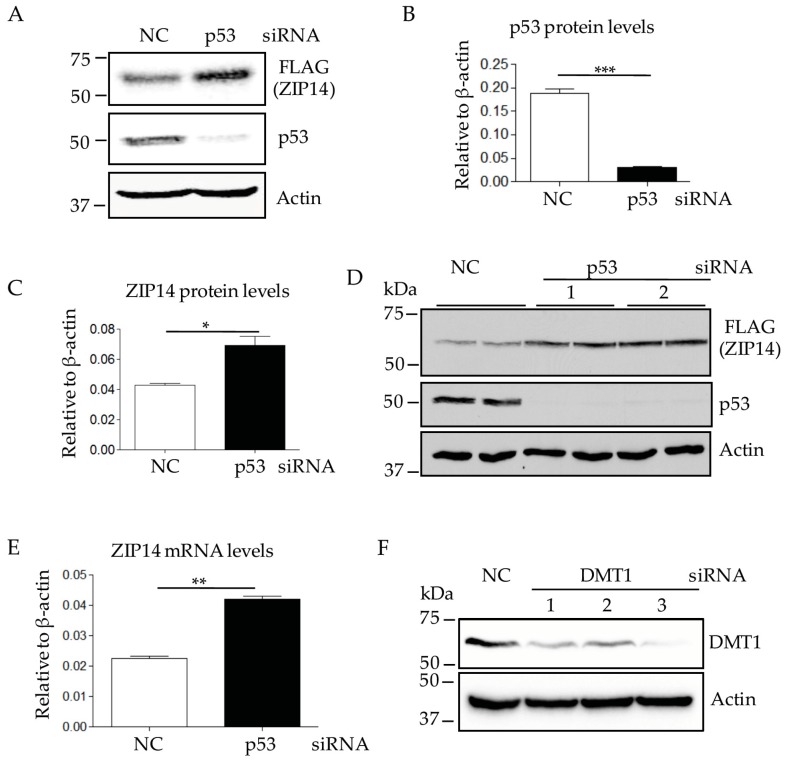

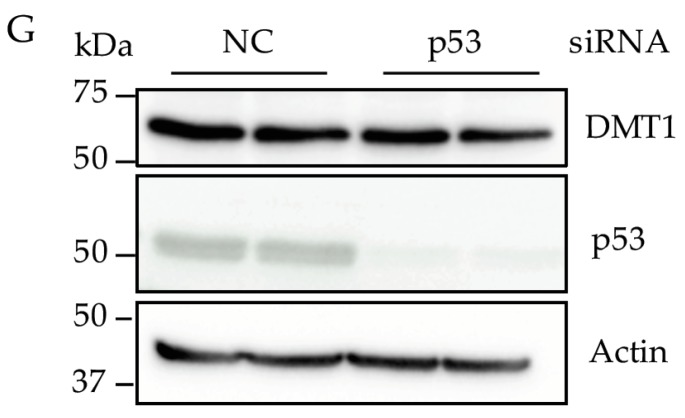
Knockdown of p53 increases endogenous ZIP14 (Zinc-regulated transporter and Iron-regulated transporter-like Protein 14) levels in human hepatoma HepG2 cells. (**A**) cells were treated with negative control (NC) siRNA or p53-targeting siRNA #1 for 48 h. Cell lysates were analyzed by immunoblotting for ZIP14 by using an anti-FLAG antibody. Blot images were visualized by Odyssey Infrared System (Licor) for quantification; (**B**–**C**) quantification of ZIP14 and p53 protein levels shown in panel A; (**D**) cells were treated with two different p53-targeting siRNAs (#1 and #2) for 48 h. Cell lysates were analyzed by immunoblotting for ZIP14. After stripping, blots were re-probed for p53 and β-actin; (**E**) ZIP14 transcript levels after p53 knockdown were quantified and normalized to those of β-actin. Experiments were repeated at least three times with consistent results; (**F**) cells were incubated with three different DMT1-targeting siRNA for 48 h. Cell lysates were analyzed by immunoblotting for DMT1. After stripping, blots were re-probed for β-actin; (**G**) cells were treated with p53-targeting siRNA #1 for 48 h. Cell lysates were analyzed by immunoblotting for DMT1. After stripping, blots were re-probed for p53 and β-actin. NC: universal scrambled negative control siRNA, * *p* < 0.05, ** *p* < 0.001, *** *p* < 0.0001, compared with control.

To examine whether changes in p53 alters ZIP14 or DMT1 levels, we chose to use the HepG2 hepatoma cell line because it expresses both ZIP14 and DMT1 [33]. Importantly, HepG2 cells express WT p53 and represent an ideal cell type to test p53 function [34,35]. A previously generated HepG2 cell line with endogenous FLAG-tagged ZIP14 was used [26,30]. This cell line keeps the endogenous gene-regulatory machinery intact and is a convenient cell model to detect ZIP14 protein by the well-validated anti-FLAG antibody [26,30]. Immunoblot analyses revealed that ZIP14 levels increased significantly after siRNA-mediated p53 knockdown (Figure 1A–C). To demonstrate the specific effect of p53 on ZIP14 protein, another p53-targeting siRNA (p53 siRNA-2) was used. Similar to p53 siRNA-1, siRNA-2 treatment efficiently reduced the expression of p53 and resulted in elevated ZIP14 levels (Figure 1D). Quantitative RT-PCR (qRT-PCR) analysis indicated that ZIP14 mRNA levels correlated with the increased ZIP14 protein when p53 expression was suppressed (Figure 1E). To immunodetect DMT1 protein in HepG2 cells, we first validated the anti-DMT1 antibody in HepG2 cells by using gene specific siRNA. Immunoblot analysis indicated that the DMT1-immunoreactive band decreases with three different siRNAs targeting DMT1, confirming the detected band migrating at 60 kDa is DMT1 (Figure 1F). In contrast to ZIP14, DMT1 did not change when p53 expression was suppressed (Figure 1G). These results suggest that the WT p53 plays a role in regulating ZIP14, but not DMT1 in HepG2 cells.

### 3.2. Knockdown of P53 Does Not Change TfR1 and Ferritin Levels in HepG2 Cells

**Figure 2:**
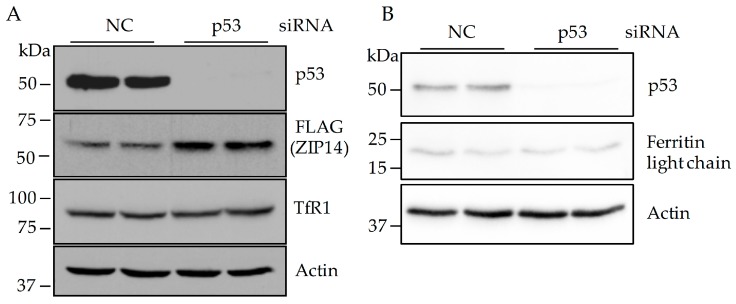

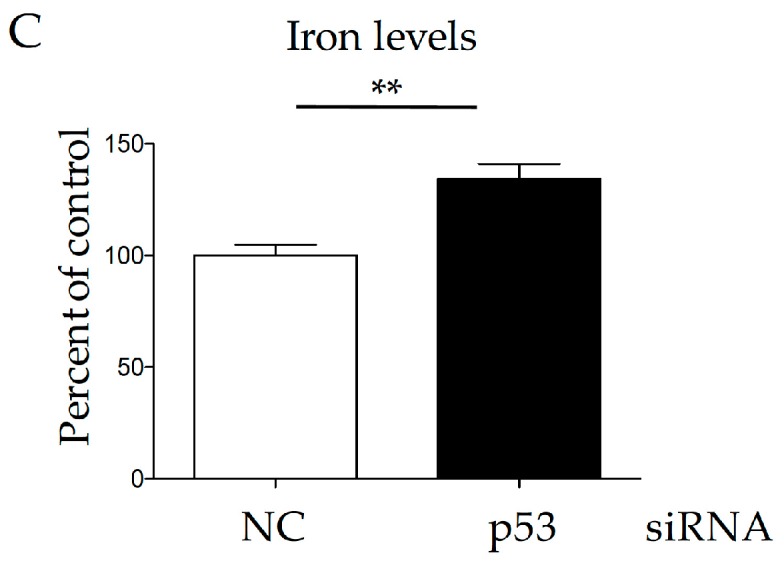
The effect of p53 knockdown on cellular iron level in HepG2 cells. HepG2-ZIP14-FLAG cells were used. Cells were incubated with negative control (NC) siRNA or p53-targeting siRNA #1 for 48 h. (**A**) cell lysates were analyzed by immunoblotting for p53. After stripping, blots were re-probed for ZIP14, TfR1, and β-actin; (**B**) to detect ferritin by immunoblot, cell lysates were incubated at 95 °C for 10 min before gel electrophoresis. Blots were first probed with anti-ferritin antibody. After stripping, blots were re-probed with anti-p53 and anti-β-actin antibodies; (**C**) cellular iron level was analyzed by Inductively Coupled Plasma Mass Spectrometry (ICP-MS). The amount of iron content was calculated as µg iron/ mg of protein. Data are normalized as percent of the iron levels of negative control siRNA transfected cells. Data represent three independent experiments. ** *p* < 0.001, compared with control.

Previous studies demonstrated that ZIP14 levels are upregulated by iron overload in HepG2 cells [30]. To examine whether the increase in ZIP14 levels after p53 knockdown results from changes in cellular iron content, we first measured protein levels of TfR1 and ferritin. TfR1 is upregulated by iron deficiency and downregulated by iron overload. In contrast, ferritin is increased in iron-loaded cells and decreased by iron depletion. Knockdown of p53 by siRNA did not significantly change the levels of TfR1 (Figure 2A) or ferritin (Figure 2B), suggesting unchanged cellular iron levels. However, unaltered ferritin and TfR1 levels could be the consequence of regulatory effects mediated by p53. For example, a study in H1299 lung cells demonstrated that p53 induction resulted in decreased TfR1 expression and increased ferritin expression [27]. To further examine the effect of p53 knockdown on cellular iron in HepG2 cells, we directly measured iron levels by ICP-MS analysis. We found that p53 knockdown resulted in a ~33% increase in cellular iron (Figure 2C), which could contribute to elevated ZIP14 when p53 expression is suppressed. These results also suggest that p53 knockdown in HepG2 cells results in no apparent changes in the levels of TfR1 or ferritin in spite of increased cellular iron.

### 3.3. Increased Expression of P53 Decreases ZIP14 in HepG2 Cells

**Figure 3:**
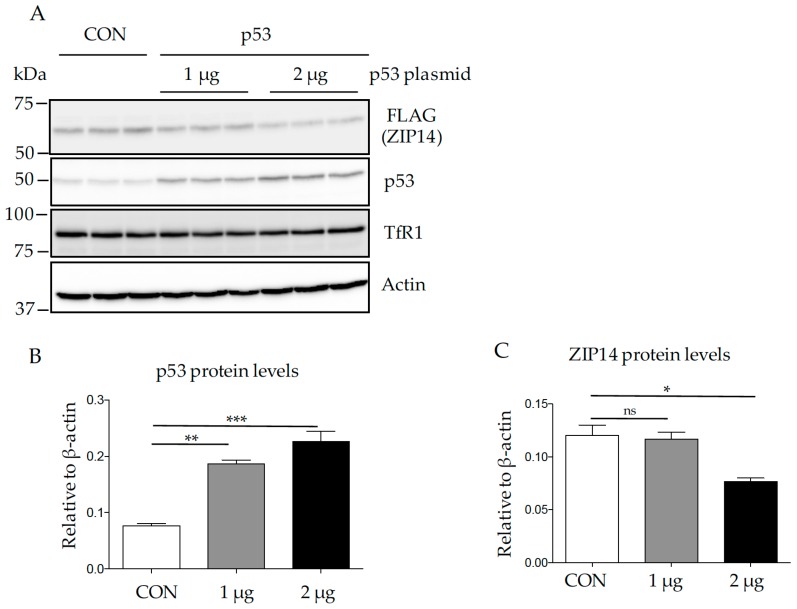

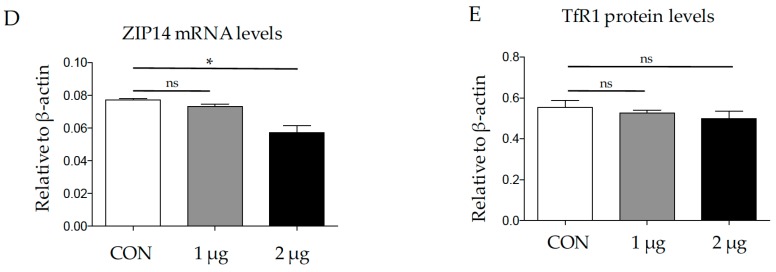
Overexpression of p53 decreases endogenous ZIP14 in HepG2-ZIP14-cells. (**A**) cells were transfected with an empty mammalian expression vector driven by human cytomegalovirus promotor (pCMV) vector (CON) or different amounts of p53- encoding vector for 72 h. Cells lysates were analyzed by immunoblotting for ZIP14, p53, TfR1 and β-actin; (**B**–**C**) quantification of p53 and ZIP14 protein levels shown in panel A; (**D**) transcript levels of ZIP14 were quantified and normalized to β-actin; (**E**) quantification of TfR1 protein levels shown in panel A. Experiments were repeated at least three times with consistent results. * *p* < 0.05, ** *p* < 0.001, *** *p* < 0.0001, ns: non-significant, compared with control.

We next tested the effect of p53 overexpression on ZIP14 in HepG2 cells by transfecting 1 or 2 µg of p53 encoding vector (pCMV-p53) into HepG2 cells for 72 h. As a control, cells were transfected with 2 µg of an empty vector (pCMV). Immunoblot analysis demonstrated that p53 protein levels increased in a dose-dependent manner with the transfected p53 plasmid (Figure 3A,B). Results showed a trend of decrease in ZIP14 levels when cells were transfected with 1 µg of p53-expressing vector and a significant decrease in ZIP14 levels (about 36%) in cells transfected with 2 µg of p53 vector (Figure 3C). Consistently, qRT-PCR analysis demonstrated that the ZIP14 mRNA levels decreased by about 26% when cells were transfected with 2 µg of p53 vector (Figure 3D). No changes in TfR1 levels were observed in cells transfected with p53 (Figure 3E), suggesting that the decrease in ZIP14 after p53 overexpression only contributed to a minor amount of total iron uptake in these cells.

### 3.4. Knockdown of Endogenously Expressed P53 Increases Endogenous ZIP14 in HEK293 ZIP14-FLAG Cells

**Figure 4:**
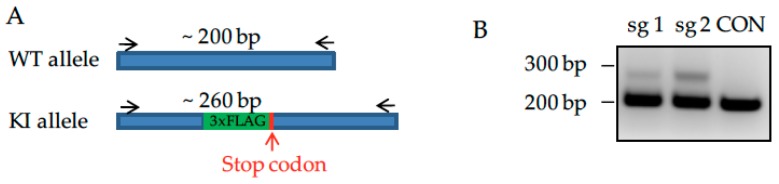

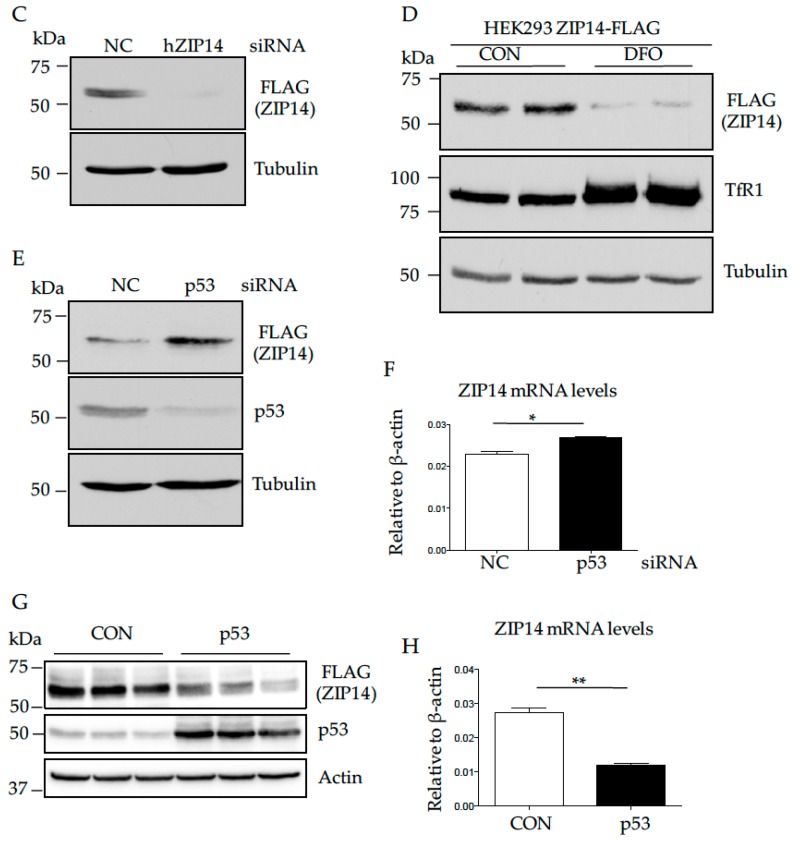
Knockdown of p53 increases endogenous ZIP14 protein level in human embryonic kidney HEK293 cells. We generated a derivative HEK293 cell line in which the endogenous ZIP14 locus was modified so as to incorporate a 3× FLAG tag at the ZIP14 C-terminus by using the Clustered Regularly Interspaced Short Palindromic Repeats (CRISPR)/CRISPR associated protein 9 (CRISPR/Cas9)-mediated genome editing approach (HEK293-ZIP14-FLAG cells). We design the single-stranded oligodeoxynucleotides (ssODN) to include about 50 bp of left and right homologous regions (HR) at each side of the ZIP14 stop codon, and the nucleotides encoding 3× FLAG sequence. (**A**) Schema for genotyping by PCR analysis: primer pair was designed to amplify a region of about 200 bp in length to cover the upstream and downstream area of ZIP14′s stop codon. After ssODN mediated homologous recombination, the knock-in allele will have codons for 3× FLAG sequence, resulting in a PCR fragment of about 260 bp; (**B**) two single-guide RNA (sgRNA) were cloned into the px330 vector. PCR results demonstrate that both sgRNAs successfully lead to the knock-in of 3× FLAG sequence in HEK293 cells. We then identified single cell clones to establish the cell line as HEK293-ZIP14-FLAG cells and these cells were used from C to H; (**C**) cells were treated with ZIP14-targeting siRNAs for 48 h. Cells lysates were analyzed by immunoblotting for ZIP14. After stripping, blots were probed for tubulin as a loading control; (**D**) to confirm that endogenously tagged 3× FLAG ZIP14 in HEK293 cells retains its biological function and regulation, cells were treated with iron chelator desferrioxamine (DFO) (100 µM) for 24 h to induce iron deficiency. Cell lysates were analyzed by immunoblotting for ZIP14. After stripping, blots were probed for TfR1 as an iron treatment control and tubulin as a loading control; (**E**) cells were transfected with p53-targeting siRNA for 48 h. Cell lysates were analyzed by immunoblotting for ZIP14. After stripping, blots were probed for p53 and tubulin; (**F**) transcript levels of ZIP14 after p53 siRNA treatment were measured and normalized to those of β-actin. Data represents three independent experiments with consistent results. NC: negative control; (**G**) cells were transfected with an empty pCMV (CON) or pCMV-p53 vector for 48 h. Cell lysates were analyzed by immunoblotting; (**H**) transcript levels of ZIP14 after p53 overexpression were measured and normalized to those of β-actin. Data represents two independent experiments with consistent results. NC: universal scrambled negative control siRNA. * *p* < 0.05, ** *p* < 0.001, compared with control.

To validate the effect of p53 on ZIP14 in another cell type, we used the HEK293 human embryonic kidney cell line. Similar to HepG2 cells, HEK293 cells express wild-type p53 [36,37]. In order to detect endogenous ZIP14 protein in HEK293 cells, the CRISPR-Cas9 mediated genome editing approach was employed to add a 3× FLAG epitope near the C-terminus just before the stop codon of ZIP14 to generate endogenous HEK 293 ZIP14-FLAG cells (Figure 4A). Cell clones containing 3× FLAG insertion were identified by PCR-based genotyping (Figure 4B). Successful in-frame insertion of the 3× FLAG epitope at the C-terminus of ZIP14 was confirmed by sequencing. Transfection of cells with ZIP14 siRNA decreased the band detected by anti-FLAG antibody (Figure 4C), indicating that we could use anti-FLAG antibody to detect endogenous ZIP14 protein in HEK293 cells. To demonstrate that adding a 3× FLAG epitope does not alter ZIP14’s regulation, we incubated cells with the iron chelator DFO to induce iron deficiency. Iron deficiency resulted in significantly lowered ZIP14 levels (Figure 4D), which is consistent with our previous results for endogenous ZIP14 in HepG2 cells and stably transfected ZIP14 in HEK293 cells [30]. These results indicate that the regulation of ZIP14 is preserved in HEK293-ZIP14-FLAG cells. We then investigated the effect of p53 suppression or overexpression on ZIP14 in these cells. Knockdown of endogenous p53 increased ZIP14 protein (Figure 4E) and mRNA levels (Figure 4F), and p53 overexpression resulted in decreased ZIP14 at both protein and mRNA levels (Figure 4G,H). These results confirm the negative regulation of ZIP14 by p53 and demonstrate that this regulation is not limited to one specific cell type.

### 3.5. P53 Suppression Increases ZIP14, Whereas P53 Overexpression Decreases ZIP14 Levels in HEK293 Cells Stably Transfected with ZIP14

**Figure 5:**
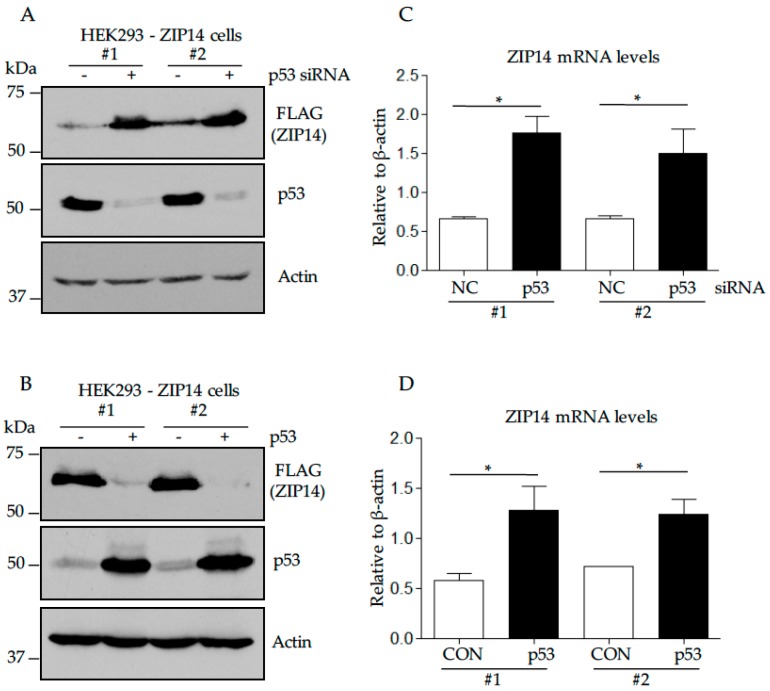
Suppression of p53 increases, whereas overexpression of p53 decreases ZIP14 levels in HEK293 cells stably transfected with ZIP14. HEK293-ZIP14-Stable cells were used in (**A**–**D**). Clone #1 and clone #2 indicate different stable cell clones; (**A**,**C**) cells were treated with negative control (NC) or p53-targeting siRNA for 48 h; (**B**,**D**) cells were transfected with empty vector (CON) or p53-encoding plasmid for 48 h. Lysates were analyzed by immunoblotting for ZIP14, p53 and β-actin (**A**,**B**). Transcript levels of ZIP14 after p53 siRNA transfection were measured and normalized to those β-actin (**C**,**D**). Data represents three independent experiments. * *p* < 0.05, compared with control.

To further examine p53’s role in regulating ZIP14 protein, we used stably transfected HEK293 cells, which express FLAG-tagged ZIP14 without its 5′ and 3′ untranslated mRNA regions (HEK293-ZIP14-Stable cells) [30]. Data obtained from two individual stable cell clones indicated that knockdown of p53 increased (Figure 5A), whereas overexpression of p53 decreased ZIP14 protein levels (Figure 5B). Interestingly, qPCR analysis by specific primers amplifying the FLAG sequence demonstrated that ZIP14 mRNA levels increased under both p53 suppression (Figure 5C) and p53 overexpression conditions (Figure 5D). Different from the endogenous ZIP14 mRNA levels in HEK293 cells (Figure 4H), the increased mRNA level (Figure 5D) observed in HEK293-ZIP14-Stable cells overexpressing p53 does not correlate with the decreased protein level (Figure 5B). This lack of correlation has been observed in our previous study when the same two cell lines were treated with iron chelator to induce iron deficiency [30]. Here, in HEK293-ZIP14-Stable cells, the decreased ZIP14 protein without a decrease in ZIP14 mRNA suggests a post-transcriptional effect of p53 on ZIP14 protein levels. These results lead us to test how p53 altered ZIP14 independently of its well-known transcriptional regulation.

### 3.6. Evidence That P53 Interacts with ZIP14 to Alter the Stability of ZIP14

**Figure 6:**
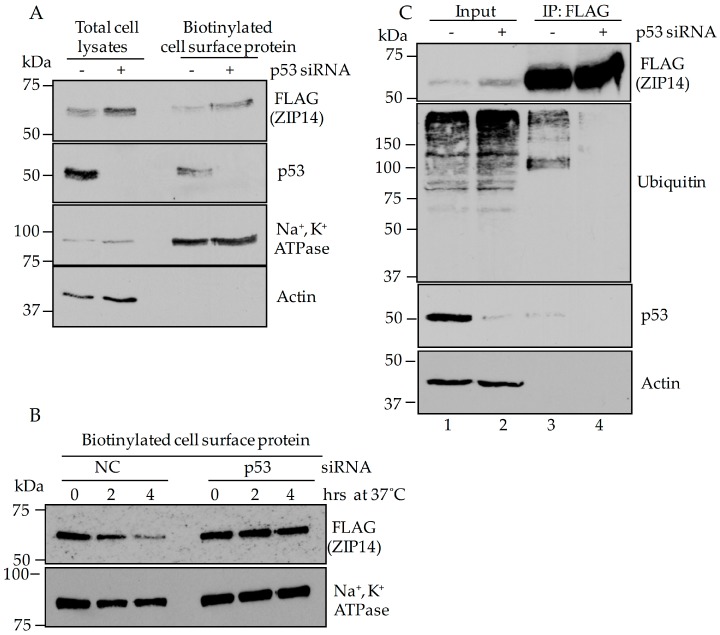
Knockdown of p53 decreases the degradation of cell surface ZIP14. (**A**) HepG2-ZIP14-FLAG cells were incubated with p53-targeting siRNA for 48 h and then cell-surface proteins were labelled with cell membrane-impermeable biotin at 4 °C for 30 min. Total cell lysates were collected and cell-surface proteins were isolated by streptavidin gel. ZIP14 levels in both the total cell lysate and cell-surface fractions were analyzed by immunoblotting; (**B**) cell-surface proteins were labeled with biotin at 4 °C and then chased for 2 and 4 h at 37 °C to allow for endocytosis. Cells were lysed and biotin-labeled cell-surface proteins were isolated by using streptavidin gel and were eluted with 50 mM DTT. Samples were analyzed by immunoblotting. Plasma membrane type Na^+^, K^+^ ATPase was used as a control for cell-surface protein; (**C**) cells were treated with p53-targeting siRNA for 48 h before the immunoprecipitation procedure. Half of the eluted fraction was probed for ZIP14 and the other half was probed with anti-ubiquitin antibody.

Previous studies demonstrated that internalized plasma membrane ZIP14 is degraded through the proteasome-mediated pathway that involves the ubiquitination of ZIP14 [30]. To further explore the mechanism underlying the regulation of ZIP14 by p53, we tested whether p53 suppression affected the stability of ZIP14. HepG2-ZIP14-FLAG cells were treated with p53-targeting or negative control siRNA for 48 h. The amount of ZIP14 on the cell surface was measured by biotinylation with an impermeable biotinylation reagent, NHS-SS-biotin. Similar to the changes in ZIP14 in the total cell lysate, cell-surface ZIP14 levels increased with p53 knockdown (Figure 6A). To test whether the increased cell-surface ZIP14 is due to decreased protein degradation, we analyzed the fate of the cell-surface ZIP14 by the biotin pulse-chase experiment. Cells were incubated with biotinylation reagent at 4 °C and then chased at 37 °C for 2 and 4 h to allow internalization. We found that knockdown of p53 inhibited degradation of cell surface-derived ZIP14 (Figure 6B). Interestingly, p53 co-immunoprecipitated with ZIP14 suggesting that they form a complex (Figure 6C, Immunoprecipitation (IP): FLAG, Western Blot (WB): p53, lane 3). Knockdown of endogenous p53 in HepG2 cells increases ZIP14 levels in the total cell lysate fractions (Figure 6C, WB: FLAG, lane 1 and lane 2), which is consistent with Figure 1A,D. The lack of difference in ZIP14 levels seen in the immunoprecipitation fractions (Figure 6C, WB: FLAG, lane 3 and lane 4) is likely due to the saturation of anti-FLAG beads by FLAG-tagged ZIP14. We observed that when similar amount of ZIP14 was pulled down by FLAG beads, the ubiquitinated ZIP14 decreased after p53 knockdown (Figure 6C, WB; Ubiquitin, lane 3 and lane 4), suggesting that p53 may play a role in regulating the degradation of ZIP14.

### 3.7. Knockdown of P53 Increases Non-Transferrin-Bound Iron Uptake in HepG2 Cells through Elevated ZIP14

**Figure 7:**
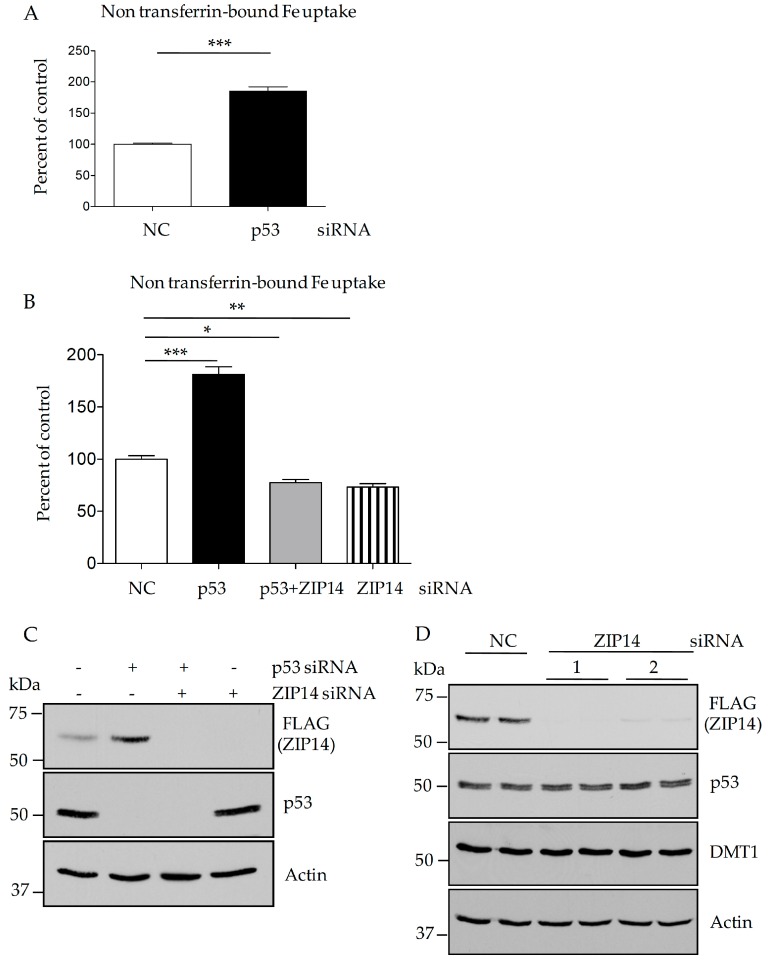
Knockdown of p53 increases iron uptake through elevated ZIP14. HepG2-ZIP14-FLAG cells were treated with p53-targeting siRNA for 48 h. (**A**) cells were incubated with 2 µM ^55^Fe-ferric-citrate for 2 h. The amount of ^55^Fe taken up by cells was calculated as cpm/mg of protein. Data are normalized as percent of the uptake of negative control siRNA transfected cells. Data represent three independent experiments; (**B**) HepG2-ZIP14-FLAG cells were treated with p53-targeting siRNA and ZIP14-targeting siRNA individually, or in combination for 48 h. Cellular iron uptake was measured after incubating with 2 µM ^55^Fe-ferric-citrate for 2 h. Data represent three independent experiments; (**C**,**D**) after siRNA treatment, cell lysates were analyzed by immunoblotting. NC: universal scrambled negative control siRNA, * *p* < 0.05, ** *p* < 0.001, *** *p* < 0.0001, compared with control.

To test the functional consequences of p53 knockdown in HepG2 cells, we evaluated the cellular iron transport by measuring NTBI uptake. We found that ^55^Fe-citrate (NTBI) uptake increased significantly after cells were incubated with p53-targeting siRNA (Figure 7A). To test whether the increased NTBI uptake is due to elevated ZIP14 levels, we performed double siRNA knockdown. We found that knockdown of ZIP14 abolished the effect of p53 suppression on cellular iron uptake (Figure 7B). Western blotting analysis validated the efficient knockdown of both ZIP14 and p53 (Figure 7C), and confirmed that ZIP14 knockdown does not alter the levels of DMT1, which is another iron import protein at the cell surface (Figure 7D). Taken together, our results in HepG2 and HEK293 cells indicate that ZIP14 is negatively regulated by p53 and suggest that ZIP14 may contribute to increased cellular NTBI uptake in p53-inactivated tumor cells.

## 4. Discussion

Nutrient uptake and metabolism in tumor cells are controlled by genetic mutations and cellular responses to the tumor microenvironment. A common metabolic phenotype of diverse tumors is enhanced nutrient uptake [38]. If the nutrient transporters, that are specifically induced in tumor cells are identified, molecules that can inhibit the pathways of their induction or block the function of the induced transporters will have significant potential as chemotherapeutic compounds. In the identified human cancers, the tumor suppressor p53 is the most frequently mutated gene. Oncogenic pathways controlling cell growth and survival are often activated by loss of p53’s proper function. Alterations in nutrient uptake and metabolic processes are also consequences of p53 mutations [39,40,41]. Elucidation of the underlying mechanisms that contribute to these alterations is critical for the understanding of tumor metabolism and for the development of cancer treatment.

As a well characterized transcription factor, p53’s function depends largely on its localization in the nucleus and its ability to trans-activate other genes causing cell cycle arrest or cell apoptosis [42]. In the present study, by using two wild-type p53 cell lines, we identified ZIP14 as a new p53-regulated protein. We found that ZIP14 is downregulated by p53 overexpression and upregulated by p53 suppression. Interestingly, the results indicate that the effect of p53 on ZIP14 protein is, at least partially independent of changes in ZIP14’s mRNA levels. Moreover, we demonstrated that p53 co-precipitates with ZIP14 and affects ZIP14’s ubiquitination and degradation. We have disclosed a new mechanism by which plasma membrane ZIP14 is regulated and highlighted the function of p53 in the regulation of cellular iron metabolism.

Our results suggest that loss of p53 may accelerate NTBI uptake through ZIP14, providing insight into the mechanisms of altered nutrient metabolism in p53-related cancers and implying potential clinical significance in patients undergoing chemotherapy. Chemotherapy is a widely used treatment for cancer patients and patients with other disorders, such as blood and autoimmune diseases [43,44]. By eliminating rapidly dividing tumor cells, chemotherapeutics also result in significantly elevated level of NTBI in the plasma [18,45,46]. Plasma NTBI is rapidly taken up by the liver, mainly through ZIP14 [47]. As a result, the extracellular NTBI will increase in the tumor microenvironment and provides a significant iron source for tumor growth, promoting therapy resistance. By using human liver hepatoma HepG2 cells, our study demonstrated that loss of p53 resulted in elevated ZIP14 levels, which increased NTBI uptake into cells, providing insight into how cells lacking p53 may acquire more iron to satiate increased growth demand when NTBI occurs within the body. Since ZIP14 can mediate the transport of other essential nutrients, including zinc and manganese [22,48], our results also suggest a potential role of ZIP14 in regulating the metabolism of these nutrients in p53-inactivated tumor cells.

Growing interest in cancer and ZIP14 has led researchers to investigate the expression level of ZIP14 in patient samples. For example, by exon array analysis, alternative splicing between exon 4A and exon 4B of ZIP14 gene was identified in colorectal tumors, and found to be regulated by the Wnt signaling pathway [49]. In this study, the authors found that exon 4A of ZIP14 was expressed about 50% lower in colorectal tumor mucosa samples than in normal samples, whereas exon 4B levels were mildly elevated in tumor samples compared to normal mucosa samples. Therefore, the authors suggested that alternatively spliced ZIP14 isoforms could be used as a cancer biomarker. A recent study, by using immunohistochemistry analysis, revealed a decreased ZIP14 expression in human prostate cancer tissues compared to that of normal prostate tissues [50]. However, the status of p53 in these patient tissue samples was not examined. Our present study provides evidence that ZIP14 is downregulated by p53. Further studies in p53-null animals and in p53-inactivated patient samples will further elucidate ZIP14’s regulation by p53.
